# A Clinical Treatise on Diseases of the Liver

**Published:** 1879-06

**Authors:** 


### BIBLIOGRAPHICAL.

A Clinical Treatise on Diseases of the Liver. By Dr. Fried. Theod. Fredricks, Professor of Clinical Medicine in the University of Berlin, etc. In three volumes. Translated by Chas. Murchison, M.D., F.R.C.P., Physician to the London Fever Hospital, etc. Wm. Wood & Co., Publishers: New York. 1879.

We have received the second and third volumes of this work, and are of the opinion that it is exhaustive of the subject. The tendency of the times is too much toward superficial reading and information. The shorter the treatise the more it is favored, as it requires less trouble to cull it. Works, however, that are, from the nature of the subject, of necessity voluminous, are passed by generally and left to the delving of sincere and persistent seekers after knowledge. On account of this fact, the work before us will probably experience a limited popularity with superficial readers. To those, however, who desire a complete, practical and scientific treatise of the physiology and pathology of the liver, combined with accurate clinical observations and deductions, these three volumes are fully calculated to give satisfaction. The publishers, Wood & Co., deserve the thanks of all interested in medical literature, for their effort to present, in a regular library series, treatises of every author whose writings may be interesting or profitable. Can we encourage and advance the enterprise by editorial reviews of each series as they are received, we will certainly do so.

